# Welding of Semiconductor Nanowires by Coupling Laser-Induced Peening and Localized Heating

**DOI:** 10.1038/srep16052

**Published:** 2015-11-03

**Authors:** Kelly M. Rickey, Qiong Nian, Genqiang Zhang, Liangliang Chen, Sergey Suslov, S. Venkataprasad Bhat, Yue Wu, Gary J. Cheng, Xiulin Ruan

**Affiliations:** 1 Purdue University, School of Mechanical Engineering, West Lafayette, IN 47907, USA; 2 Purdue University, Birck Nanotechnology Center, West Lafayette, IN 47906, USA; 3 Purdue University, School of Industrial Engineering, West Lafayette, IN 47907, USA; 4 Los Alamos National Lab, Center for Integrated Nanotechnology, Division of Materials Physics and Application, Los Alamos, NM 87545, USA; 5 Qatar Foundation, Qatar Environment and Energy Research Institute, Doha, Qatar; 6 SRM Research Institute, SRM University, Chennai, 603203, India; 7 Department of Chemical and Biological Engineering, Iowa State University, Ames, IA 50010, USA

## Abstract

We demonstrate that laser peening coupled with sintering of CdTe nanowire films substantially enhances film quality and charge transfer while largely maintaining basic particle morphology. During the laser peening phase, a shockwave is used to compress the film. Laser sintering comprises the second step, where a nanosecond pulse laser beam welds the nanowires. Microstructure, morphology, material content, and electrical conductivities of the films are characterized before and after treatment. The morphology results show that laser peening can decrease porosity and bring nanowires into contact, and pulsed laser heating fuses those contacts. Multiphysics simulations coupling electromagnetic and heat transfer modules demonstrate that during pulsed laser heating, local EM field enhancement is generated specifically around the contact areas between two semiconductor nanowires, indicating localized heating. The characterization results indicate that solely laser peening or sintering can only moderately improve the thin film quality; however, when coupled together as laser peen sintering (LPS), the electrical conductivity enhancement is dramatic. LPS can decrease resistivity up to a factor of ~10,000, resulting in values on the order of ~10^5^ Ω-cm in some cases, which is comparable to CdTe thin films. Our work demonstrates that LPS is an effective processing method to obtain high-quality semiconductor nanocrystal films.

Highly efficient photovoltaic (PV) cells and batteries are increasingly important as energy demands increase, and nanocrystal (NC) films have the potential to alleviate such demands. When nanocrystals are very small, multiple exciton generation (MEG) can occur due to the quantum confinement effect, and this is desirable for photovoltaic applications^1^. Nanocrystals are also promising for lithium-ion battery applications for their enormous surface area^2^. However, organic surfactants are usually needed to form high-quality NCs during synthesis, and they hamper electron and heat transport between nanocrystals by forming an insulating shell around them. Such problems hinder them from achieving the hypothesized high efficiency or pose significant thermal management challenges. Processing methods are needed to improve the nanocrystal contact while preventing nanocrystal growth, preserving nanocrystal morphology, and maintaining the porous structure of the film.

Prior studies have indeed demonstrated that heat treatments can improve the thin film quality and raise the efficiency^3^,^4^,^5^. Drndic, *et al.* annealed CdSe quantum dots at various temperatures between 110° and 430 °C, which yielded increased film conductivity^3^. They attributed this to the decomposition of electrically insulating trioctylphospine oxide (TOPO), as well as the improved physical QD contact^3^. Gur, *et al* sintered both CdSe and CdTe nanorod (NR) films, increasing their quantum efficiency^4^, while Olson, *et al* showed higher light absorption and a ~5% power conversion efficiency in CdTe nanorod Schottky devices after heating the film to 400 °C in a CdCl_2_ atmosphere^5^. Another study specifically showed crystallinity improvements on individual CdTe nanowires (NWs)^6^. These studies show that heat-treating nanofilms are promising for PV applications. However, although high-temperature heat treatments vaporize/decompose surfactants, they increase particle size, diminishing a film’s quantum confinement effect.

An alternative treatment is laser-based sintering^7^,^8^, where the ultrafast heating may enhance NC physical contact without significantly affecting the nanoparticle size. Surfactants cannot withstand locally high temperatures, and therefore they decompose. Simultaneously, NCs do not significantly grow during such a brief time period. They remain largely unchanged in size and could maintain their quantum confinement effect. Recently, pulsed laser annealing was applied to process colloidal nanocrystal films into interconnected nanostructures^9^,^10^,^11^. However, previous pulsed laser sintering studies focused on thin-film CdTe^7^ or on NCs generally at or above the melting point^8^, whereas we aim to achieve a smaller degree of fusion. Also, electronic transport properties after treatments were rarely reported^10^,^11^, making it hard to assess the effectiveness of the treatments. Several other new nanoscale processing methods have been demonstrated to weld nanocrystals while preserving the original size, morphology, and pores. These include the plasmonic welding of silver nanowires^12^ and lithium-assisted electrochemical welding in silicon nanowires^13^. However, the plasmonic mechanism is limited to metal nanowires, and the purpose of the film was to act as PV electrodes, rather than the PV film itself^12^. The lithium-assisted approach is limited to the lithium battery environment. It is clear that further innovative efforts on processing methods and understanding their effects on the electrical transport properties are needed to weld semiconductor nanocrystals.

In this work, we take a step further on the previous pulsed laser sintering studies and demonstrate that coupled laser peening and sintering can achieve high quality nanoporous films. We choose to focus on nanowires rather than shorter nanocrystal morphologies because nanowires have larger aspect ratios that can provide longer, uninterrupted paths for charge transport. The key innovation of our approach is to find out the coupling effect of the two processes, i.e., performing a laser peening compaction step followed by pulsed-laser sintering. The films treated under the two-step LPS process show two to four orders of magnitude improvement in electrical conductivity compared to either laser sintering or laser peening alone. We attribute this drastic enhancement to the fact that the laser peening can generate controlled pressure to densify the porous thin film and bring non-contacting NWs into good contact; following this, pulsed laser sintering locally melts the contact regions and fuses those nanowires together. Neither step alone can generate optimum outcomes. One could always use higher sintering power to achieve lower resistivity, rather than perform a compression phase beforehand. However, such high power will result in the wires completely melting, and the general NW morphology and grain boundaries would be lost. This compression phase allows the sintering power applied afterwards to be low enough to improve contact through grain boundary fusion instead. It provides a new, efficient, and scalable laser processing method for fabricating high-quality nanoporous thin films from wet-synthesized colloidal nanocrystals, and it can have broad implications for emerging applications such as electronics, photovoltaics, thermoelectrics, battery electrodes, etc.

## Experiments

**Figure 1:**
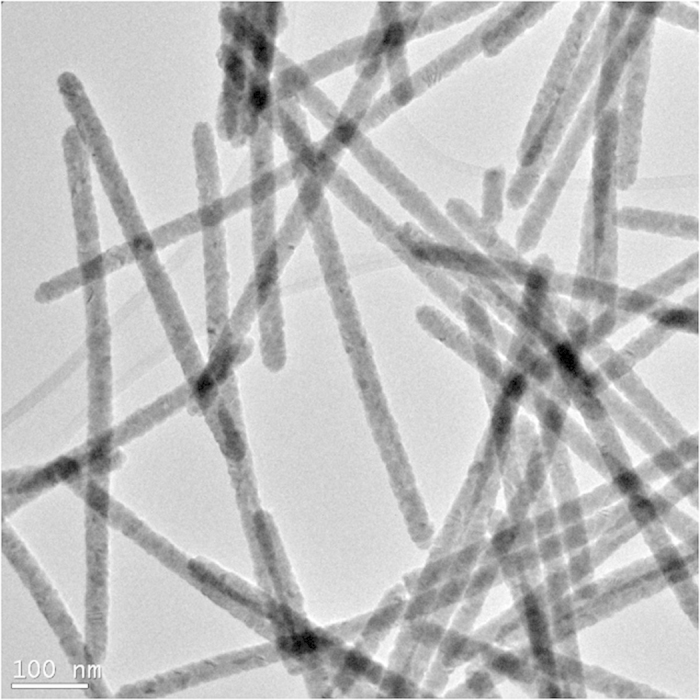
TEM image of CdTe nanowires. The wires were produced using a tellurium precursor of TeO_2_, ethylene glycol, PVP40, and KOH combined with a Cd precursor of CdCl_2_ and ethylene glycol. Average diameter: ~40 nm. Wires range from 400 to 900 nm in length, for an estimated average length of ~680 nm.

We first synthesized the NWs using a method similar to that of Zhang, *et al.*^14^. The X-ray diffraction data for the wires is similar to JCPDS card No. 75–2083, indicating cubic CdTe (See Supplementary Figure S2 online). A TEM image is shown in Fig. 1.

**Figure 2:**
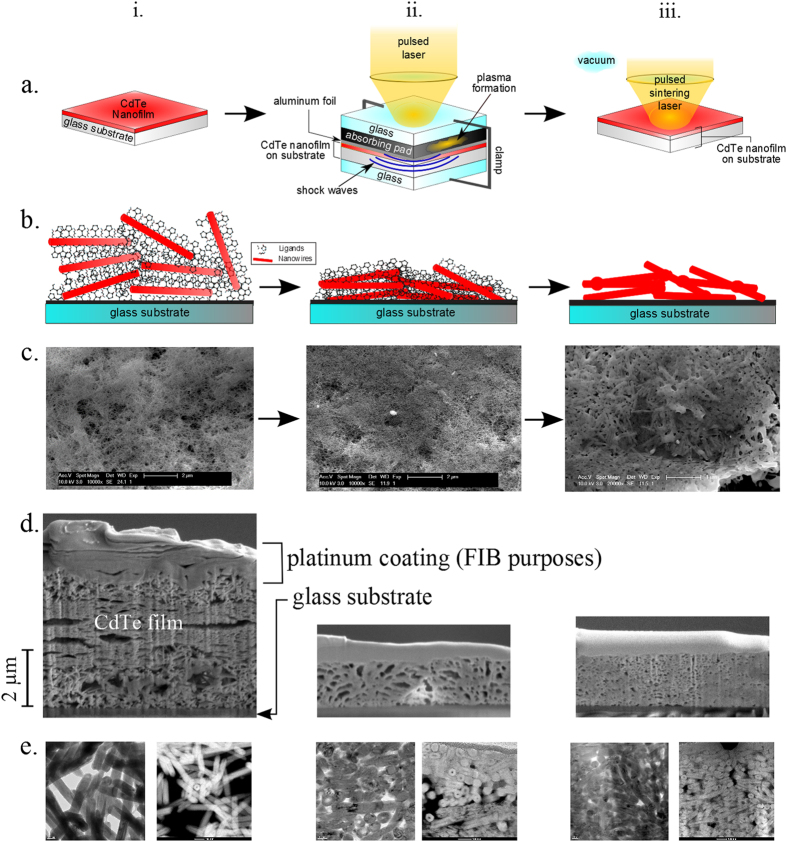
Detailed schematic of the entire laser peen sintering process. (**a**) (i) NW film is untreated. (ii) Film undergoes the first stage of processing, the laser peen/shock process. The CdTe substrate is placed between another glass surface and a black-body absorbing material (graphite). A piece of glass is then put on top of the graphite pad to contain it. Next, the laser is shot at the transparent top glass layer in ~5 ns pulses. Plasma is generated from the graphite pad. In turn, shock waves hit the CdTe film in pulses. (iii) Film then undergoes sintering in a separate laser facility. (**b**) (i) Solution-deposited nanowire films, where nanowires are wrapped with ligands and unconnected; (ii) compressing the film brings the nanowires in better contact; (iii) sintering can remove ligands and strongly join nanowires by forming chemical bonds between them, while general morphology of nanowires are preserved. (**c**) Top-view SEM images of a CdTe NW film (i) before any treatment^16^; (ii) after 400 MPa has been applied, and no sintering afterwards^16^; (iii) after 400 MPa peening and then sintering with two 25-ns pulses. The density of the film greatly increases and the porosity decreases. Note: we have obtained permission from *SPIE* to reprint images from Figs. 3ci and 3cii. We originally used these in our previous work, reference 16. (**d**) cross-sectional pieces extracted from a film using the focused ion beam (FIB) technique. (i) untreated; (ii) compressed at 400 MPa; (iii) compressed at 400 MPa and then sintered at 24 mJ/cm^2^ with two 25 ns pulses. The film thickness and porosity dramatically decrease with treatments. The top coating is platinum, which is layered on the film to assist in the FIB process. These images were cropped from original FIB images for space and clarity purposes. Furthermore, the FIB procedures used to obtain the cross-sectional images were performed on samples *after* their electrical conductivities were measured. In this way, the platinum and other factors involved in the FIB process did not affect the electrical conductivity measurements. (**e**) TEM images showing the NW connections after various treatments. (i) untreated; (ii) compressed at 400 MPa, and no sintering afterwards; (iii) compressed at 400 MPa and later sintered.

After a CdTe NW film (~5 μm) was uniformly deposited onto a large glass substrate, different sections of it were separated. Some pieces underwent treatment at a given pressure and/or laser power, while others were left untreated. Each piece (Fig. 2a (i)) was taken from the same NW substrate in order to ensure that all data were taken from the same film with the same density, quality, age, etc. The pressurizing mechanism is illustrated in Fig. 2a (ii), and the process is as follows: the substrate is sandwiched between a hard glass surface and aluminum foil. On top of the aluminum foil is a confined, black-body absorbing material, graphite. On top of this is another piece of glass to confine the entire system. A Continuum® Surelite^TM^ YAG laser (wavelength = 1024 nm) is then shot towards the graphite, sponge-like pad, which generates plasma (Fig. 2a (ii)). As the plasma is generated, it remains confined between a top layer of glass and the foil. The foil, which remains in its solid state, pushes on the film-on-glass-substrate below. In this way, high-pressure shock waves hit the film and compress it without changing its temperature. The laser power determines the heat absorbed by the patch, how much it expands, and therefore the pressure it applies to the film^15^. The pressure is applied rapidly, i.e. one 5 ns pulse of beam exposure. This is known as laser peening, or laser “shocking.” The resulting film is much thinner and denser after this process, as diagrammed in Fig. 2b (ii). The entire film is scanned in this manner, so that all areas are treated uniformly.

In the pulsed laser-sintering process, which is performed after the laser shock process, the film is radiated by an excimer laser (wavelength of λ = 248 nm, pulse duration = 25 ns) in a vacuum chamber (Fig. 2a (iii)). Again, the entire film is scanned so that the whole surface is treated evenly. The laser beam size is fixed at 0.5 cm^2^. During pulse laser heating, the local electromagnetic field enhancement is generated around the contacted regions between the nanowires, resulting in fusion of the nanowires without completely melting them. It is noted here that both laser shock compaction and pulsed heating are important to the final 3D “connected” NWs structure. Only when laser shock compaction brings the NWs into “linked” position, can the pulsed heating fuse the NWs together by localized heating without completely melting them. When the process is complete, the wires are fused together, as illustrated in Fig. 2b (iii).

## Results and Discussion

SEM images (Fig. 2c) show the morphology of a CdTe NW thin film before laser treatment (i)^16^, after laser peening (400 MPa) (ii)^16^ and after both laser peening (400 MPa) and laser sintering (iii), respectively. After laser peening, films are compacted and denser, but remain very porous. It is the localized heating around the contacting NWs during pulsed-laser heating that allows the NWs to fuse together without changing their nanoscale geometry (Fig. 2c, iii).

Cross-section images were also collected and analyzed, as shown in Fig. 2d. Totally untreated (uncompressed, unsintered) films have thicknesses over 4 μm (Fig. 2d (i)); they contain pores larger than 600 nm, and the wires are not well-connected. On the other hand, the same film compressed at 400 MPa reveals a thickness only 1/3 of the untreated sample, with smaller pores (2d (ii)). Finally, a sample from the same film shock-compressed at 400 MPa and then sintered at 14 mJ/cm^2^ has the densest structure (2d (iii)). The pores are even smaller than in the other two cases, as the wires have been welded together. The film is slightly thicker than the solely compressed sample’s; this is likely because upon treatment, the wire diameters slightly expand from the heat and inflate the film.

**Figure 3:**
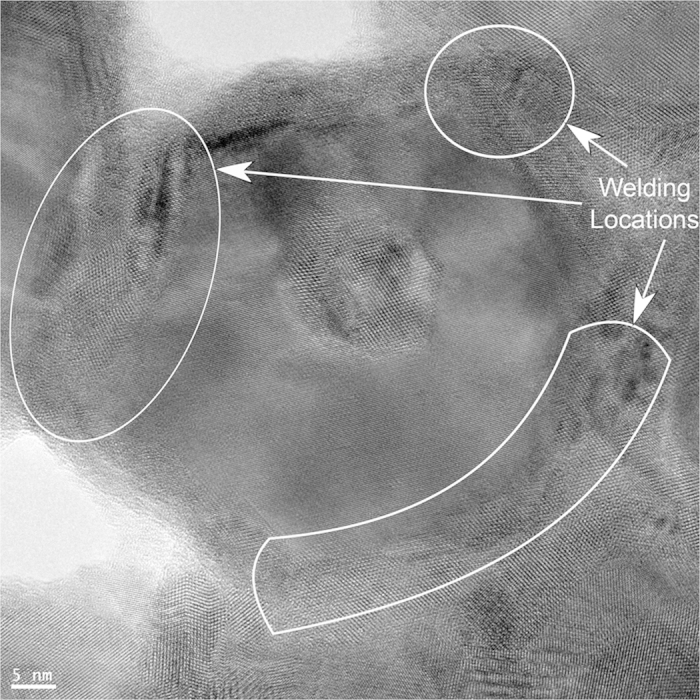
HRTEM image of NW film sample after compression and sintering. The lattices of adjacent wires have welded, yet their diameters have changed little, remaining at a range of 25–50 nm. The marked areas indicate welding sites.

To illustrate the wire connections up close, TEM images were taken (Fig. 2e). Initially, the wires overlap but do not show any clear welding (Fig. 2e (i)). During shock-compression, NWs are compressed together, closing gaps and aiding in improved NW contact while maintaining their shape and boundaries (2e (ii)). After sintering, the wires have welded together and individual boundaries are more difficult to discern; however, they are still present and the diameters have not increased substantially (2e (iii)). A close-up of such welding can be seen in Fig. 3, where the wires’ boundaries have melded together. This improved contact contributes to increased charge transfer, while such shape retention may promote quantum confinement if these treatments are performed on thinner nanowires in the future.

**Figure 4:**
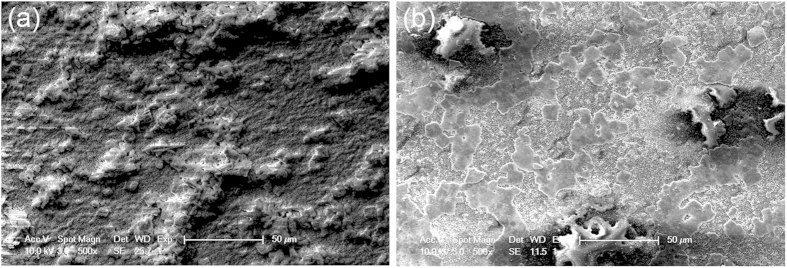
SEM images of untreated and treated films. Low-magnification view of an untreated CdTe NW film (**a**) and a film that has been compressed at 400 MPa, then sintered at 20 mJ/cm^2^. (**b**) Scale bar = 50 nm.

On the macroscale, the wires tend to cluster together and form larger, individual groups when sintered, an effect which has been observed in previous film sintering studies^17^. The film “tears” as some nanocrystals meld closer to each other and break away from others. Had our film been very thin or sparse, this gap formation would have caused a loss in connection across the film; however, because our film has a high starting volume fraction, the lowest layers of CdTe appear to be less affected by the heat, and they tend to maintain the previous structure. The result is that, throughout many areas of the film, upper layers form clusters of welded wires while lower, less-treated layers keep a connection between these high-conductivity groups (Fig. 4). Overall, this structure yields much higher conductivities than untreated films. How to precisely control the gap growth is not covered in this paper, but is something that shall be studied in the future.

Element analysis on the samples was performed to ensure that the film did not oxidize during treatment. XPS data shows that oxidation (TeO_2_) is only present on the film surface and decreases with film depth (see Supplementary Information section online) for both sintered and unsintered films. Although there is a difference in the oxidation percentage between sintered and unsintered samples, the oxide levels remain well below 10% (molar basis) for both.

**Figure 5:**
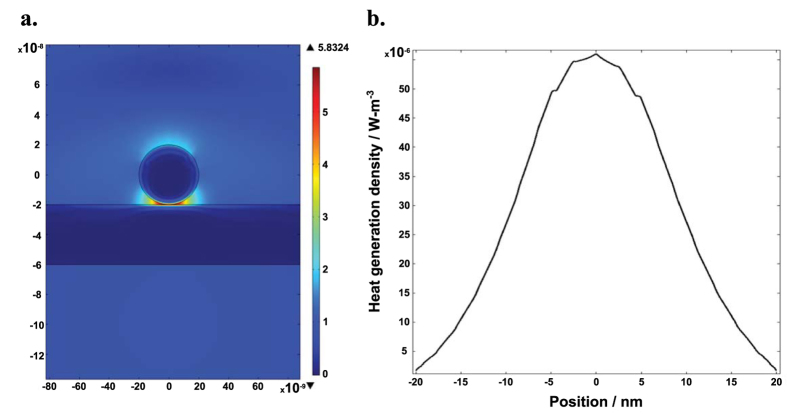
Simulation results of the laser sintering process. (**a**) Laser beam local field concentration simulated by Comsol Multiphysics® with a Gaussian electromagnetic wave as incident beam on a cross of nanowire junctions. The Gaussian beam laser was delivered with an electrical field of 1 V/m to nanowires that were suspended in air, touching each other, and crossing at ninety degrees. (**b**) The heat generation density as a function of distance to the contact point, described by the power loss density calculated in Comsol Multiphysics®.

The morphology results point to the possibility of junction-specific welding during laser sintering. This behavior, if true, is beneficial for forming high quality thin films while maintaining the individual particle morphology. To help assess this possibility, we have performed electromagnetic modeling with Comsol Multiphysics®. To set the model, a Gaussian beam laser was delivered to nanowires with an electrical field of 1 V/m, where nanowires suspended in air crossed at ninety degrees touched each other. Each nanowire was modelled as having a circular cross-section with a diameter of 40 nm and dielectric function taken from references 18 and 19. It can be seen in Fig. 5a that the local electrical field was concentrated as high as 5.8 V/m near a given junction area, generating a “hot spot” at this contact point. In order to further explore the heating process generated by these “hot spots,” the heat generation density versus X-axis position was plotted in Fig. 5b. The heat generation density was calculated with the Comsol Multiphysics® power loss density, which is determined by multiplying the illumination power density by the nanostructure’s absorption coefficient. The majority of heat generation was observed around a junction-like area ±10 nm from the contact point, supplying sufficient contact-specific fusion. Farther away from the contact point, the heat generation density decreases. These results confirm that heat generation is much higher at the contact, which enables the contact-specific fusion.

**Figure 6:**
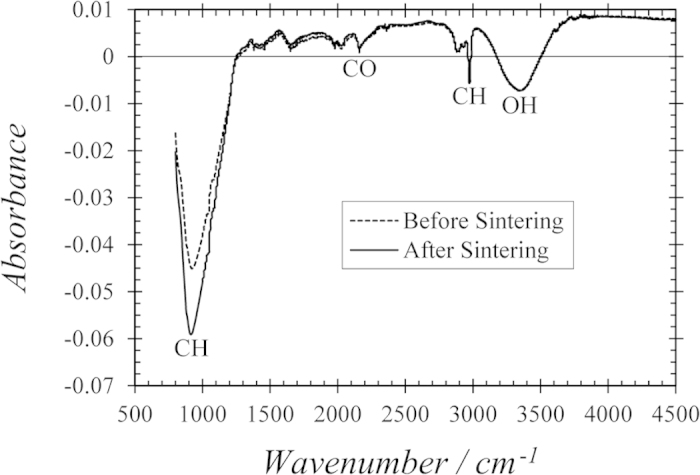
FTIR absorbance spectra. Spectra for a 400 MPa-compressed, unsintered film and a 400 MPa-compressed, sintered film.

To characterize the properties of our thin films, Fourier transform infrared spectroscopy (FTIR) was performed on two samples (again, each from the same film): one that was sintered and one that was not. The data in Fig. 6 reveal little difference between the sintered and unsintered samples, and that all ligands (represented by CH and CO groups), water, and hydrazine hydrate levels (both represented by OH groups) are low before as well as after sintering. This indicates that most ligands were washed away during the centrifuging and dip-coating processes, which are performed before any treatments. Thus, an increase in electrical transport after sintering is likely the result of improved wire contact due to increased density and wire melding, rather than the decomposition of insulating ligands or evaporation of OH groups.

Once each sample was treated at various conditions and top views were imaged via SEM, the in-plane conductivities at several different parts of each sample were measured using a 2-probe configuration. On the untreated samples, the resistances were found to linearly increase with channel length, indicating low contact resistance between film and electrodes and thus little contribution from it to the overall, measured film resistance. The average resistivity, ρ, for each sample was obtained by averaging the dark current data from different locations on that sample (For details, see the Supplementary Information). The ρ error for each sample incorporates the standard deviation of these measurements at different locations. It is large at 50→90%; however, it remains within the appropriate order of magnitude that illustrates a vast ρ reduction. Furthermore, the spacing between electrodes is large enough to cover a large number of nanowires. Therefore, we believe our sample is statistically uniform in the microstructure.

**Figure 7:**
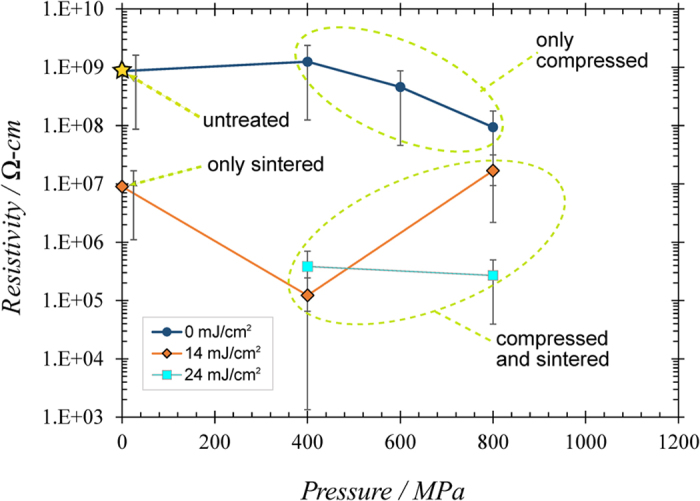
Film resistivity vs. pressure. Film resistivity as a function of the pressure that was applied during the laser peening phase. Data shown are for samples that are unsintered, sintered at 14 mJ/cm^2^, and sintered at 24 mJ/cm^2^.

The effects of compression pressure and sintering energy are shown in Fig. 7. The average resistivity ρ for each sample is graphed vs. the compression pressure. Some samples only underwent the compression phase (dark blue circles), while another sample underwent only sintering (labeled). The rest were compressed and later sintered. Each of these sample’s ρ was compared to the average untreated ρ (all untreated samples had ρ values ~10^9^ Ω-cm), which is indicated by a star. It is apparent that LPS’d films are much more conductive than totally untreated or partially treated films. For instance, the samples that are compressed and not later sintered exhibit ρ decreases no larger than a factor of 10 from the untreated. On the other hand, the sample that was sintered without compression beforehand showed a larger improvement of 100, but even this pales in comparison to samples that are dually treated (one could heavily increase the sintering power to lower ρ; however, this lower ρ would result from the film melting rather than improved NW proximity and contact area fusion. As discussed in the Introduction, we do not wish to liquefy anything, because the general nanowire-based film morphology would not be maintained). The dually treated samples exhibit ρ’s up to 10^4^ times lower than the untreated ρ. The vast decrease can be attributed to two factors: (a) the lower porosity and higher density of the films, and (b) the way in which the wires are more effectively fused together once this density has increased. This allows a continuous pathway for charge while maintaining the morphology.

**Figure 8:**
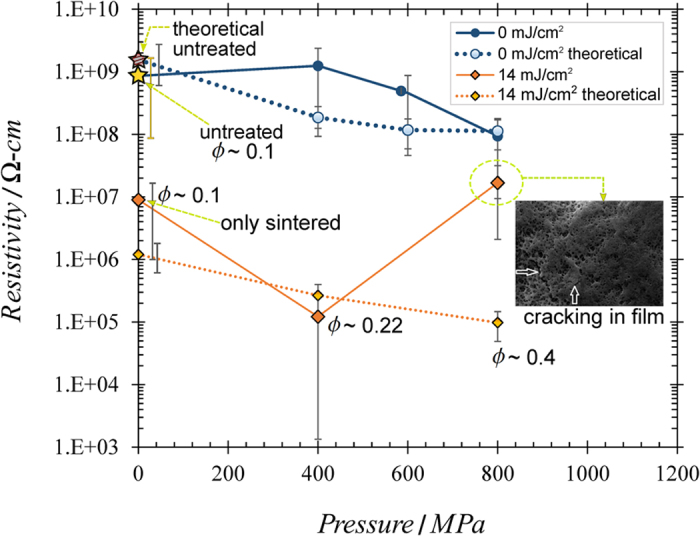
Measured film resistivity compared with theoretical resistivity, as a function of pressure applied during the laser peening phase. Measured resistivity values and theoretical values based on percolation theory are shown. For clarity, values are shown only for the unsintered and 14 mJ/cm^2^-sintered samples. The volume fraction ϕ is shown next to some data points to convey the increase in this value as the compression pressure increases. The inset image shows the cracks that were found in an 800 MPa compressed, 14 mJ/cm^2^-sintered film^16^. This indicates that the high compression may cause fatigue in the film. This fatigue may cause the film to crack once it is sintered^16^. These cracks may be the reason this sample has a much higher resistivity than the theoretical prediction^16^. Note: we have obtained permission from *SPIE* to reprint the inset image in this figure. We originally used it in our previous work, reference 16.

It is also clear that higher sintering energies, on average, result in lower ρ’s; simultaneously, higher compression values also tend to result in lower ρ’s. This is obvious in single-phased treated films (compression only: dark blue circles; sintering only, first point on 14 mJ/cm^2^ curve), but these trends often hold true for dually treated films as well. For example, the ρ’s of compressed, 24 mJ/cm^2^-sintered samples are lower, on average, than the ρ’s of compressed, 14 mJ/cm^2^-sintered samples. Furthermore, ρ decreases with higher compression values for the 24 mJ/cm^2^-sintered film. However, sintering and compression trends do not *always* apply for the dually treated samples. For example, the compression trend is not consistent for the 14 mJ/cm^2^-sintered samples. One would expect the 800 MPa-compressed sample to have a lower ρ than the 400 MPa-compressed sample. Instead, it is >100 times higher. This is likely due to the cracks that formed in the film. We saw this on a preliminary sample treated at the same conditions (Fig. 8, inset)^16^. The higher 800 MPa pressure may have fatigued the film, and this fatigue allowed cracks to form once the film was sintered; this would explain its higher resistivity^16^. The 24 mJ/cm^2^-sintered sample, however, does not have a higher ρ at 800 MPa. This is likely due to the following: higher compressions yield thinner films. When such a thin film is sintered, it is easier for it to “tear” as nanoparticles distance themselves from some neighbors in order to weld to others (Fig. 4). This cluster-forming fatigues the film and causes cracking in various locations. If the sintering power is high enough, however, the stronger fusion it creates between wires within the clusters can make up for lost conductivity caused by any gaps outside. Thus, any ρ increase caused by cracking is well countered by ρ decreases resulting from stronger wire fusion in other locations along the channel. More research will need to be performed in this area to understand this balance.

Despite some inconsistencies, it is clear that *both* compression and sintering are needed to considerably improve charge transfer and simultaneously maintain film morphology. The resulting lower ρ’s are comparable to single and polycrystalline CdTe thin films at ~10^5^ Ω-cm^7^,^20^,^21^,^22^,^23^.

To understand the electrical resistivity data, we performed calculations based on the classical percolation theory. The elaborated details are given in the Supplementary Information, and here we only provide a summary. In this theory, “sticks” (nanotubes & nanowires) randomly distributed throughout an insulating medium yield a film condutivity σ that is proportional to *(N-N*_*c*_)^*t*^, where *N*_*c*_ is the minimum wire density required to achieve conductivity from one side of the film to the other, *t* is the critical exponent, and *N* is the actual wire density within the film^24^,^25^,^26^. This trend can be described by the following equation, where particle aspect ratio *a* = *L/d* is considered^27^:


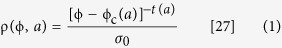


Here, ρ is the film resistivity (=1/σ), ϕ is the actual nanowire volume fraction within the structure; ϕ_c_ is the minimum nanowire volume fraction required to achieve conduction from one electrode to the other, and *t*(*a*) is the critical exponent, which accounts for the structure’s dimension and percolation model type^26^,^28^. The conductivity constant σ_o_ depends on both the resistivity ρ_nt_ of the individual nanowire material and the average conductivity between wires at intersection sites^27^. Our wires are “hard-core” wires, which represents the realistic situation where wires *overlap* each other at intersection points. In order to compare our results to theory, we first looked at films that were solely compressed and unsintered. The theoretical values, determined from equation (1), are shown in Fig. 8 (blue circles, dotted). Each experimental result is within an order of magnitude of the theoretical value, and thus our compression results reasonably reflect percolation theory applied with the known constants. Using lower σ_0_ and ϕ_c_ values for the 14 mJ/cm^2^-sintered sample estimates (once the wires are heated and their surfaces begin melding together, the wires can no longer be considered “hard-core”), we obtain much lower theoretical ρ values, which are mapped in Fig. 8 (orange diamonds, dotted). Experimental resistivities for the uncompressed and 400 MPa samples sintered at 14 mJ/cm^2^ compare well with theoretical numbers, differing by less than an order of magnitude. The 800 MPa, 14 mJ/cm^2^ sample, however, exhibits a much higher ρ than what the theory predicts. This is likely because of cracks that formed in the sample (inset), which the theory does not account for. Overall, our results match well with percolation theory, and we believe it is an appropriate way to predict the resistivities of our films.

## Conclusion

The synthesis, treatment, and characterization of CdTe NW films undergoing coupled laser peening and sintering were described. Morphological, spectral, and electrical analyses were performed on the films before and after treatment, and the results were compared. LPS results in denser, better-connected CdTe films with resistivities up to 10,000× lower, and it is vastly more effective than compression or pure sintering treatment alone. The multiphysics simulations that couple electromagnetic (EM) and heat transfer modules demonstrate that during pulsed laser heating, local EM field enhancement is generated specifically around the contact areas between two semiconductor nanowires, producing localized heating. Percolation theory has been used to mathematically explain our resistivity results in terms of film density. This paper has demonstrated the fundamental mechanism of LPS and analyzed the resulting compact nanowires network on the electrical properties of the CdTe NW films. The presented method shows much improvement in electrical properties and has the potential to benefit the development of high quality, solution-synthesized and nanomaterial-based electro-optical devices in the future.

## Methods

### CdTe Nanowire Synthesis

Cadmium telluride wires were synthesized using a method similar to that of Zhang, *et al.*^14^. Tellurium dioxide (TeO_2_), polyvinylpyrrolidone (PVP40), ethylene glycol and potassium hydroxide (KOH) were mixed to make a tellurium precursor. After one hour of stirring and heating at 140 °C, a cadmium chloride (CdCl_2_)/ethylene glycol mix (Cd precursor) was added. The formula was mixed for another hour at 140 °C. The resulting wires had diameters ranging from of 25 to 50 nm (average ~40 nm) and were 400–900 nm in length (average ~675 nm). The wires were precipitated and rinsed in a centrifuge twice with deionized water, then twice with 200-proof ethanol. They were deposited onto glass substrates under nitrogen via dip coating with hydrazine hydrate (80%) and acetonitrile.

### Microscopy

The TEM image in Fig. 1 was acquired using an FEI Tecnai TEM. The images shown in Figs. 2d, 2e and 3 were gathered using an FEI Titan 80–300 keV Field Emission Environmental Transmission Electron Microscope—Scanning Transmission Electron Microscope. All remaining SEM images were acquired using an FEI XL40 SEM.

### Electrical Resistivity Characterizations

Once each film sample was treated at various conditions and imaged via SEM, aluminum electrodes were deposited via thermal evaporation to aid in electrical conductivity measurements. Thin bands of CdTe were left uncoated so that the conductivity could be measured across the channels. Silver paste was dropped onto the electrodes at either side of each channel so that the probes of the electrical measurement system would not scratch the electrode surfaces.

The in-plane conductivities at several different parts of each sample were measured using a 2-probe configuration. On the untreated samples, resistances were found to linearly increase with channel length, indicating low contact resistance between film and electrodes and thus little contribution from it to the overall, measured film resistance. The high film resistance was confirmed using Van der Pauw measurements, where the film resistance across the configuration was too high to be discerned. Thus, any improvements in conduction after treatments were due to changes across the film itself rather than lower contact resistance. The testing voltage ranged from −2 V to +2 V, and dark currents were recorded. Average resistivities for each sample were calculated using the dark current data, average film thicknesses, and channel lengths.

To some degree, measured resistivity values (ρ) depend on the film quality and testing location. The film is a porous mesh of randomly distributed nanowires, and nanowires may be better connected in some areas than others, even after treatment. For this reason, multiple areas of each sample were tested, and their dark currents averaged over comparatively large channel dimensions. The film thickness of each sample was also averaged. The ρ error incorporates such differences from one location to another on a given film. Furthermore, some samples were fabricated at different times; however, both were made in the same manner from NW batches synthesized using the exact same processes, and so have similar structures. Nevertheless, any error from this is also incorporated into our uncertainty. It is large at 50→90%; however, it remains within the appropriate order of magnitude that illustrates a vast ρ reduction. Furthermore, the spacing between electrodes is large enough to cover a large number of nanowires. Therefore, we believe our sample is statistically uniform in the microstructure.

## Additional Information

**How to cite this article**: Rickey, K. M. *et al.* Welding of Semiconductor Nanowires by Coupling Laser-Induced Peening and Localized Heating. *Sci. Rep.* **5**, 16052; doi: 10.1038/srep16052 (2015).

## Supplementary Material

Supplementary Information
