# Supplementary material for: Welding of Semiconductor Nanowires by Coupling Laser-Induced Peening and Localized Heating
Source: Sci Rep. 2015 Nov 3;5:16052. doi: 10.1038/srep16052 (PMC4630610; doi:10.1038/srep16052)
Supplement: Supplementary Information [file srep16052-s1.pdf]

Supplementary Information for

Welding of Semiconductor Nanowires by Coupling Laser-Induced Peening and Localized Heating

Kelly M. Rickey[1,2], Qiong Nian[2,3], Genqiang Zhang[4], Liangliang Chen[1,2], Sergey Suslov[2,5], S. Venkataprasad Bhat[1,2,6], Yue Wu[7], Gary J. Cheng[1,2,3], Xiulin Ruan[1,2,*]

[1]Purdue University, School of Mechanical Engineering, West Lafayette, IN 47907, USA
[2]Purdue University, Birck Nanotechnology Center, West Lafayette, IN 47906, USA
[3]Purdue University, School of Industrial Engineering, West Lafayette, IN 47907, USA
[4]Los Alamos National Lab, Center for Integrated Nanotechnology, Division of Materials Physics and Application, Los Alamos, NM 87545, USA
[5]Qatar Foundation, Qatar Environment and Energy Research Institute, Doha, Qatar
[6]SRM Research Institute, SRM University, Chennai, 603203, India
[7]Department of Chemical and Biological Engineering, Iowa State University, Ames, IA 50010, USA

*corresponding author: ruan@purdue.edu

Additional Results & Discussion

XPS

Element analysis was performed on the samples to ensure that the film did not oxidize during treatment. XPS data shows that oxidation (TeO_2) is only present on the film surface and decreases with film depth for both sintered and unsintered films, shown in Supplementary Fig. S1. Although there is a difference in the oxidation percentage between sintered and unsintered samples, the oxide levels remain well below 10% (molar basis) for both samples. The levels are documented in Supplementary Tables S1 and S2.

XPS RESULTS

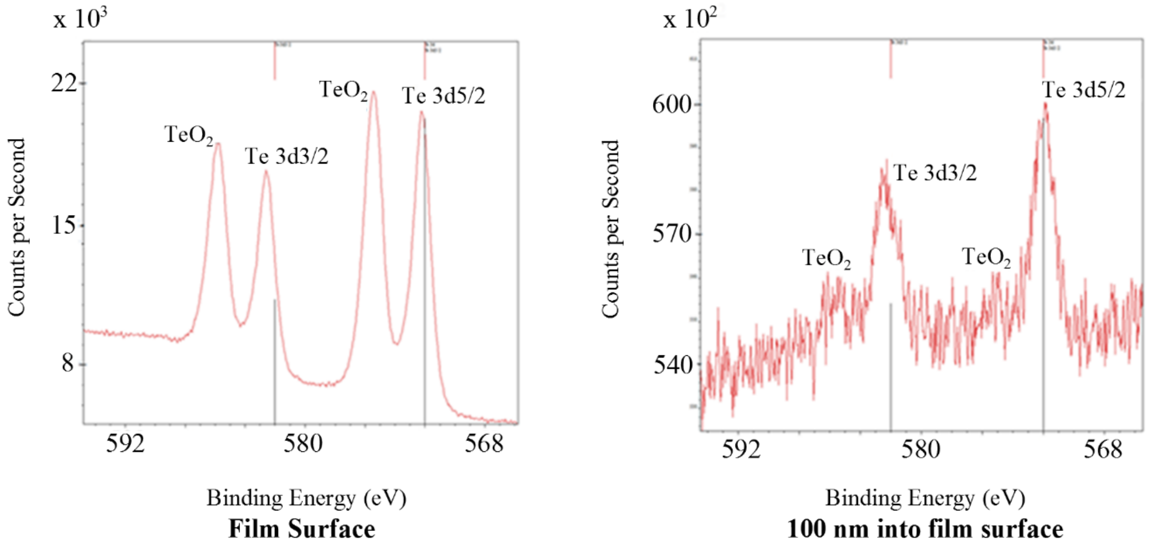

Supplementary Figure S1. **XPS results.** XPS data on (a) the surface of CdTe film and (b) 100 nm below the surface of the same film. XPS process performed by Dmitry Zemlyanov.

Per mol of film material:

depth [nm]	Te [mol]	Cd [mol]	Oxide [mol]	O from Water or Hydrazine Hydrate [mol]
0	0.272	0.194	0.066	0.467
0.6	0.268	0.214	0.071	0.447
30.6	0.170	0.172	0.028	0.629
90.6	0.084	0.134	0.013	0.770

Per 1 gram of film material:

depth [nm]	Te mass [g]	Cd [g]	Oxide [g]	O from Water or Hydrazine Hydrate [g]
0	0.534	0.335	0.016	0.115
0.6	0.514	0.361	0.017	0.108
30.6	0.421	0.375	0.009	0.195
90.6	0.279	0.394	0.005	0.322

Supplementary Table S1. **Untreated CdTe NW film compound content.** The top table shows the element amount per mol of film material. The bottom table shows the element amount per 1 g of film material.

Per mol of film material:

depth [nm]	Te [mol]	Cd [mol]	Oxide [mol]	O from Water or Hydrazine Hydrate [mol]
0	0.275	0.271	0.131	0.323
0.6	0.320	0.287	0.119	0.274
30.6	0.415	0.426	0.069	0.090
90.6	0.301	0.650	0.047	0.002

Per 1 gram of film material:

depth [nm]	Te mass [g]	Cd [g]	Oxide [g]	O from Water or Hydrazine Hydrate [g]
0	0.482	0.419	0.029	0.071
0.6	0.515	0.406	0.024	0.055
30.6	0.513	0.463	0.011	0.014
90.6	0.342	0.651	0.007	0.0003

Supplementary Table S2. **Treated (LPS'd) CdTe NW film compound content.** The top table shows the element amount per mol of film material. The bottom table shows the element amount per 1 g of film material.

Electrical Resistivity Details

Electrical resistivity characterizations

Once each sample was treated at various conditions and imaged via SEM, aluminum electrodes were deposited via thermal evaporation to aid in electrical conductivity measurements. Thin bands of CdTe were left uncoated so that the conductivity could be measured across the channels. Silver paste was dropped onto the electrodes at either side of each channel so that the probes of the electrical measurement system would not scratch the electrode surfaces.

The in-plane conductivities at several different parts of each sample were measured using a 2-probe configuration. On the untreated samples, resistances were found to linearly increase with channel length, indicating low contact resistance between film and electrodes and thus little contribution from it to the overall, measured resistance. The high film resistance was confirmed using Van der Pauw measurements, where the film resistance across the configuration was too high to be discerned. Thus, any improvements in conduction after treatments were due to changes across the film itself rather than lower contact resistance. The testing voltage ranged from -2 V to +2 V, and dark currents were recorded. Average resistivities for each sample were calculated using the dark current data, average film thicknesses, and channel lengths.

To some degree, measured resistivity values (ρ) depend on the film quality and testing location. The film is a porous mesh of randomly distributed nanowires, and nanowires may be better connected in some areas than others, even after treatment. For this reason, multiple areas of each sample were tested, and their dark currents averaged over comparatively large channel dimensions. The film thickness of each sample was also averaged. The ρ error incorporates such differences from one location to another on a given film. It is large at 50→90%; however, it remains within the appropriate order of magnitude that illustrates a vast

ρ reduction. Furthermore, some samples were fabricated at different times; however, both were made in the same manner from NW batches synthesized using the exact same processes, and so have similar structures. Finally, the spacing between electrodes is large enough to cover a large number of nanowires. Therefore, we believe our sample is statistically uniform in the microstructure.

Percolation Theory-Based Calculations & Estimates

To understand the electrical resistivity data, we performed calculations based on the classical percolation theory. In this theory, "sticks" (nanotubes & nanowires) randomly distributed throughout an insulating medium adhere to the following relation[1-2]:

$$\sigma \ \propto \ (N - N_c)^t \tag{1}$$

where σ is film conductivity, N_c is the minimum wire density (per area for a 2-D film; per volume for a 3-D film) required to achieve conductivity from one side to the other, and N is the actual wire density within the film[1-3]. Often, this wire density is expressed as a volume fraction—i.e., the fraction of total volume taken up by nanowires. Furthermore, the effect of wire aspect ratio $a = L/d$ must be considered[4]:

$$\rho\,(\phi, a) = \frac{[\phi - \phi_c(a)]^{-t(a)}}{\sigma_0} \tag{2}$$

where ρ is the film resistivity (=1/σ), ϕ is the actual nanowire volume fraction within the structure; ϕ_c is the minimum nanowire volume fraction required to achieve conduction from one electrode to the other, and $t(a)$ is the critical exponent, which accounts for the structure's dimension and percolation model type[1,5]. It is also dependent on the aspect ratio a. For hard-core, randomly distributed nanowires of aspect ratio a = 17, this value ~ 1.8[4]. The conductivity constant σ_o depends on both the resistivity ρ_{nt} of the individual nanowire material

(in our case, CdTe) and the average conductivity between wires at intersection sites[6]. It can be estimated from the following equation[4]:

$$\sigma_o \approx \frac{1}{\rho_{nt}\ a^{2-t}} \tag{3}$$

where t = 1.8 and a = 17 for our network. Assuming that the contact resistance between CdTe objects averages ~10^7 **Ω**-cm[6–12], equation (3) results in a theoretical value of 5.7×10^{-8} S/cm.

According to numerical simulations previously performed by Xue, as well as Foygel *et al*, ϕ_c is ~0.1 for "soft-core" wires of finite width (i.e., L/d < 100) and aspect ratio = 17[4,13]. A soft-core wire intersects another by physically passing through it at the crossing site. However, our wires are "hard-core" wires. This represents the realistic situation where wires *overlap* each other at intersection points. To our knowledge, no extensive volume fraction studies specific to randomly distributed, hard-core wires exist. However, it is known that perfectly aligned, hard-core shapes require ϕ_c to be no larger than 0.16, regardless of aspect ratio[3,4]. Given that aligned wires typically require higher ϕ_c than randomly oriented wires[3,4,14], we can contend that our wires do not require ϕ_c to be larger than 0.16. Indeed, Foygel *et al* have been able to obtain good estimates for hard-core wires with a >> 1. In such cases, ϕ_c equals the following[4]:

$$\phi_c \approx \frac{0.6}{a} \tag{4}$$

With an average aspect ratio of a = 17, our ϕ_c value is ~ 0.035. In general, fewer wires per volume are required for electrode-to-electrode conduction if they are randomly oriented, have large a, and have a hard-core structure[3,4,13,14].

This information can explain our resistivity results. To find ϕ for our samples, we used ImageJ software[15] to analyze the top and cross-sectional views of the untreated section and estimated the average nanowire density per area. We considered the fact that such images are only 2-D, and from this, estimated the starting volume fraction to be ~ 0.1. From this, we calculated the volume fraction of treated sections by looking at those film thicknesses and

comparing them to the untreated thickness. Although these ϕ values are just estimates, it is obvious that each value is much greater than the required volume fraction of $\phi_c \sim 0.035$ needed for electrode-to-electrode conduction. This explains how we are able to achieve conduction through the channel even before any treatments.

In order to compare our results to theory, we first looked at films that were solely compressed and unsintered. The ϕ value increases from 0.1 up to ~0.4 for a film treated at 800 MPa. These values, along with $t = 1.8$, $a = 17$, $\phi_c = 0.035$, and $\sigma_0 = 5.7 \times 10^{-8}$ S/cm were plugged into equation (2) to obtain the theoretical ρ values for compressed but *unsintered* films, shown in Fig. 8 (blue circles, dotted). We then plotted them alongside the actual ρ results (blue circles, solid) for comparison. Each result is within an order of magnitude of the theoretical value, and thus our compression results reasonably reflect percolation theory applied with the known constants.

Equation (2) also explains why the resistivity decreases so dramatically with treatment: ϕ increases with compression. Meanwhile, ϕ_c remains at ~ 0.035. The increasing difference, $\Delta\phi$, between the two values is then taken to a power of $-t$, which remains unchanged at ~ -1.8 [4]. Thus, ρ decreases exponentially as $\Delta\phi$ increases, and $\Delta\phi$ increases with progressive treatments. The theoretical values of the 14 mJ/cm^2-sintered samples were also estimated. This time, we used a different value for σ_0. Once the wires are heated and their surfaces begin melding together, the wires can no longer be considered "hard-core." Rather, they are something in between hard and soft wires. In this case, σ_0 should take on a smaller value because the contact resistance between wires is smaller. We understand that the wires are not completely melting together to form bulk CdTe. However, at the points of intersection, where the wires are fused together, we approximate no contact resistance. Thus, an estimation of bulk CdTe resistivity *at these junctions* is used. The bulk value used was on the order of 10^4 Ω-cm, the lowest resistivity we could find for polycrystalline bulk CdTe thin films[10]. This

lowered the ρ_{nt} value from 10^7 in the unsintered cases to 10^4 for the sintered cases. Thus, the σ_0 value increased from 5.7×10^{-8} S/cm to 5.7×10^{-5} S/cm. Furthermore, because the wires are somewhat soft and do not completely overlap each other anymore, we decreased the required volume fraction ϕ_c from 0.0353 to 0.001, based on Foygel *et al*'s assessments[4]. These changes in constants led to a decrease in the overall theoretical ρ values given by equation (2), which are mapped in Fig. 8 (orange diamonds, dotted).

Resistivities for most of these samples compare well with theoretical numbers, differing by less than an order of magnitude. The 800 MPa, 14 mJ/cm^2 sample, however, exhibits a much higher ρ than what theory predicts. This is likely because of cracks that formed in the sample (inset), which theory does not account for.

Overall, our results match well with percolation theory, and we feel it is an appropriate way to predict the resistivities of our films. There is certainly error involved in some of the constants chosen; nevertheless, the theory qualitatively explains our results.

XRD graphs for CdTe Nanowires

The XRD data for our nanowire films, shown in Supplementary Fig. S2, is similar to JCPDS card No. 75 – 2083, indicating cubic CdTe[16-18].

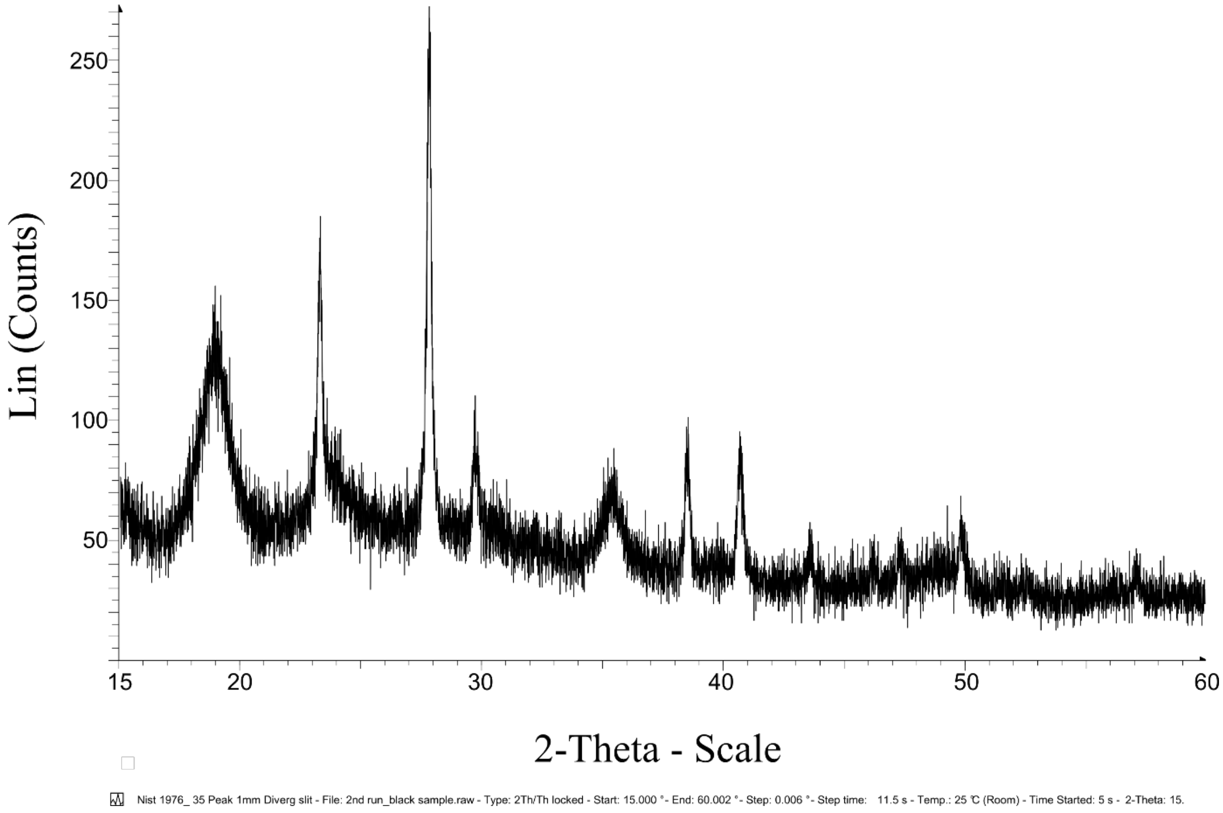

Supplementary Figure S2. **XRD data for CdTe nanowire film**.

References for Supporting Information

1. Stauffer, D. *Introduction to Percolation Theory*. (Taylor & Francis, Inc., 1985).

2. Madaria, A. R., Kumar, A., Ishikawa, F. N. & Zhou, C. Uniform, highly conductive, and patterned transparent films of a percolating silver nanowire network on rigid and flexible substrates using a dry transfer technique. *Nano Res.* **3,** 564–573 (2010).

3. Pike, G. & Seager, C. Percolation and conductivity: A computer study. I. *Phys. Rev. B* **10,** 1421–1434 (1974).

4. Foygel, M., Morris, R., Anez, D., French, S. & Sobolev, V. Theoretical and computational studies of carbon nanotube composites and suspensions: Electrical and thermal conductivity. *Phys. Rev. B* **71,** 104201 (2005).

5. Hu, L., Hecht, D. S. & Grüner, G. Percolation in Transparent and Conducting Carbon Nanotube Networks. *Nano Lett.* **4,** 2513–2517 (2004).

6. Bobrova, E. A. & Klevkov, Y. V. Deep electron levels in undoped polycrystalline CdTe annealed in liquid Cd. *Semiconductors* **45,** 865–871 (2011).

7. Hatanaka, Y., Niraula, M., Aoki, Y., Aoki, T. & Nakanishi, Y. Surface processing of CdTe compound semiconductor by excimer laser doping. *Appl. Surf. Sci.* **142,** 227–232 (1999).

8. Dzhafarov, T. D., Yesilkaya, S. S., Yilmaz Canli, N. & Caliskan, M. Diffusion and influence of Cu on properties of CdTe thin films and CdTe/CdS cells. *Sol. Energy Mater. Sol. Cells* **85,** 371–383 (2005).

9. Picos-Vega, A. *et al.* Cd self-doping of CdTe polycrystalline films by co-sputtering of CdTe–Cd targets. *J. Appl. Phys.* **83,** 760 (1998).

10. Chu, T. L. Thin film cadmium telluride solar cells by two chemical vapor deposition techniques. *Sol. Cells* **23,** 31–48 (1988).

11. Chung, G. Y., Park, S. C., Cho, K. & Ahn, B. T. Electrical properties of CdTe films prepared by close-spaced sublimation with screen-printed source layers. *J. Appl. Phys.* **78,** 5493 (1995).

12. Fluck, R. A. & Jaffe, M. J. Improvement of the productivity in the growth of CdTe single crystal by THM for the new PET system. in *2007 IEEE Nucl. Sci. Symp. Conf. Rec.* **410,** 1783–1787 (IEEE, 2007).

13. Xue, Q. The influence of particle shape and size on electric conductivity of metal–polymer composites. *Eur. Polym. J.* **40,** 323–327 (2004).

14. Du, F., Fischer, J. & Winey, K. Effect of nanotube alignment on percolation conductivity in carbon nanotube/polymer composites. *Phys. Rev. B* **72,** 121404 (2005).

15. Rasband, W. S. *ImageJ*. Available at <http://imagej.nih.gov/ij/>. (Accessed: February 2014)

16. Wageh, S., Higazy, A.A., Algradee, M. Optical Properties and Activation Energy of A Novel System of CdTe Nanoparticles Embedded in Phosphate Glass Matrix. *J. Mod. Phys.* **02,** 913–921 (2011).

17. Tiwari, A. K., Verma, V. K., Jain, T. A. & Bajpai, P. K. Conclusive Growth of CdTe Nanorods by Solvothermal Decomposition Using Single Source Precursors. *Soft Nanosci. Lett.* **03,** 52–57 (2013).

18. Wu, Y., Zhang, G., Fang, H., Yang, H. (Purdue University), U.S. Patent Application. *Number US 13/891,914*. (2013).
